# High admission glucose levels increase Fas apoptosis and mortality in patients with acute ST-elevation myocardial infarction: a prospective cohort study

**DOI:** 10.1186/1475-2840-12-171

**Published:** 2013-11-15

**Authors:** Jing Chang, Gong Zhang, Li Zhang, Yuan-Ping Hou, Xiu-Lan Liu, Lin Zhang

**Affiliations:** 1 Heart Failure Center, Department of Cardiology, Chao-Yang Hospital, Capital Medical University, Beijing, China; 2 Department of Internal Medicine, Chao-Yang Hospital, Capital Medical University, Beijing, China

**Keywords:** Myocardial infarction, Glucose, Apoptosis, Mortality

## Abstract

**Background:**

The presence of diabetes and plasma glucose concentration on admission are associated with adverse outcomes after an acute myocardial infarction (AMI), as high glucose can induce vascular endothelial cell apoptosis. This study explored the relative associations among admission plasma glucose level, soluble Fas (sFas) concentration, and long-term survival in patients with acute ST-elevation myocardial infarction (STEMI).

**Methods:**

This prospective cohort study include 83 patients with acute STEMI. Based on their admission plasma glucose levels (7.8 and 11.1 mmol/L as the limits for low and high levels, respectively), patients were allocated into one of three groups: normal glucose (n = 33), median glucose (n = 24), and high glucose (n = 26). The admission plasma level of sFas was measured with a sandwich enzyme-linked immunosorbent assay (ELISA). Patients were followed up for an average of 89 ± 20 months for all causes of death and cardiovascular death.

**Results:**

sFas levels were significantly higher in the high glucose group compared to the normal glucose group (5.87 ± 1.70 mmol/L vs. 3.07 ± 0.93 mmol/L, respectively, P < 0.05). The sFas level was positively associated with the admission plasma glucose level. The correlation coefficient (*R*) was 0.747, and *R*^2^ was 0.559. Mortality was significantly higher in the high glucose group compared to the normal glucose group (19.2% vs. 3.0%, respectively, P < 0.05).

**Conclusions:**

In patients with acute STEMI, plasma glucose level was high on admission, and sFas apoptosis levels were increased. Long-term follow-up revealed that a high admission plasma glucose level was associated with higher mortality compared to a normal admission glucose level.

## Background

Acute myocardial infarction (AMI) is a major cause of mortality throughout the world. Electrocardiography can be used to classify AMI as an ST elevation myocardial infarction (STEMI) or as a non-ST elevation myocardial infarction (non-STEMI) [1]. More than three million patients are reportedly diagnosed with STEMI each year [2]. The in-hospital mortality of STEMI patients in the national registries of the European Society of Cardiology countries varies from 6% to 14% [3].

There are many clinical trials and animal studies on AMI. Several investigators have described apoptosis after AMI in humans. Recent studies suggest that Fas apoptosis system activation may play a pathogenic role in AMI and therefore may be a valuable diagnostic tool [4-6].

Diabetes is an independent predictor of mortality after AMI [7]. However, some patients with AMI are unaware that they have abnormal glucose metabolism, have not undergone an oral glucose tolerance test, and do not have a diagnosis of diabetes. The relationship between admission plasma glucose level and AMI prognosis needs to be studied. Some studies have recently reported that STEMI patients with high glucose at admission are at an increased risk of 30-day and 1-year mortality [8].

The poor prognosis of patients who are diabetic or who have a high glucose level is related to many factors. Myocardial cell apoptosis is one possible reason, and the Fas/Fas ligand (Fas/FasL) complex may play a role in the process. In the present study, we initially examined the relationship between the admission plasma glucose level and soluble Fas (sFas) concentration, then we investigated the effect of different admission plasma glucose levels on mortality.

## Methods

### Experimental design and subjects

This prospective cohort trial was carried out at the Heart Center of the Chao-Yang Hospital at the Capital Medical University (Beijing, China). The research protocol was reviewed and approved by the Medical Ethics Committee of the Capital Medical University Chao-Yang Hospital. All patients provided informed consent to participate after they were informed of risks associated with the study verbally and in writing. Male and female patients (older than 18 years) were selected for participation based on the following criteria: chest pain lasting 30 min or longer, ST elevation greater than 0.1 mV in at least two contiguous precordial leads, or at least two adjacent limb leads on the electrocardiogram or evidence of new onset left-bundle branch block. Patients were excluded for the following reasons: previous myocardial infarction, valvular heart disease, autoimmune disease, infective disease, tumor, or serious liver or kidney dysfunction (i.e., serum creatinine level greater than 170 μmol/L or an alanine aminotransferase level greater than three times the normal upper limit).

### Patient allocation

**Figure 1:**
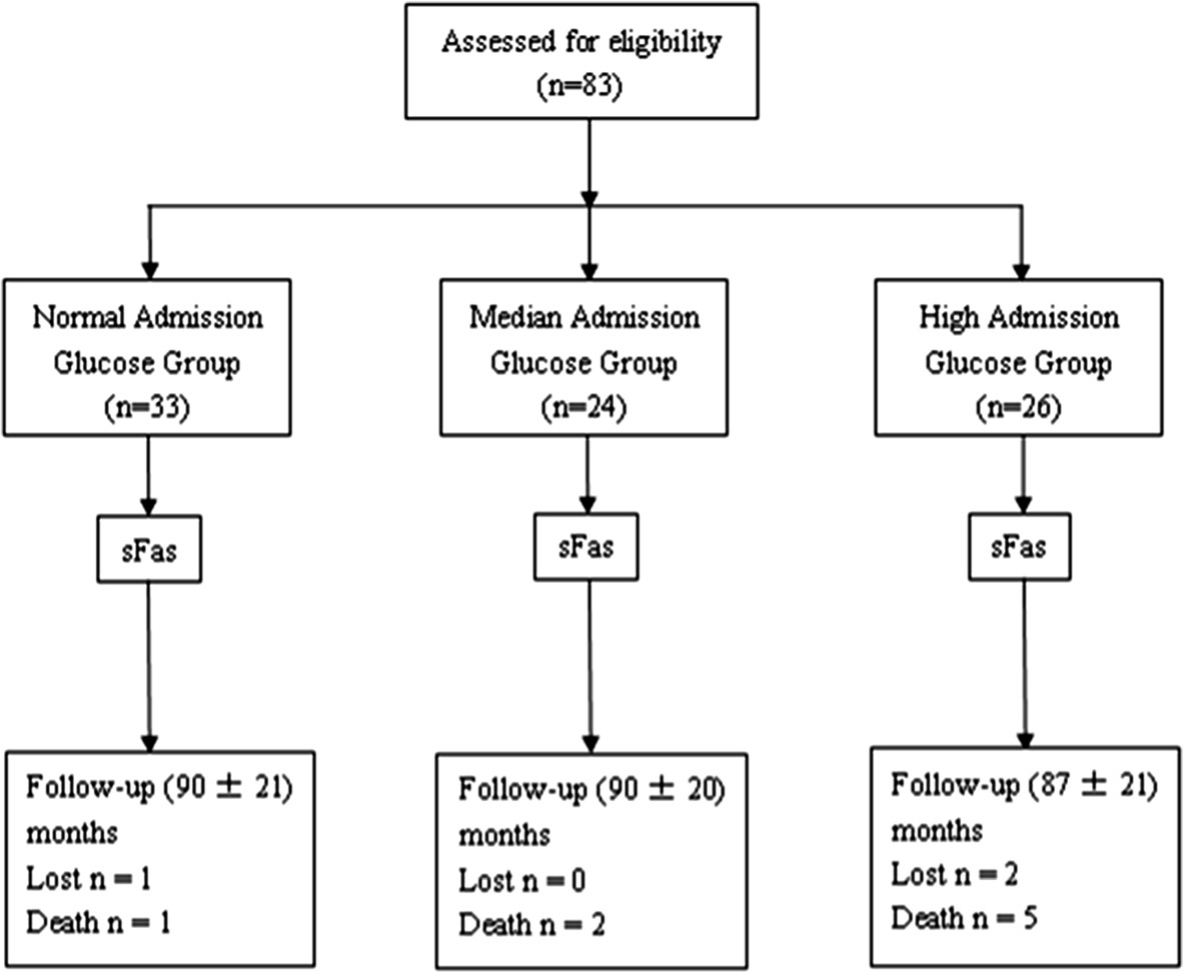
**Patient flow diagram.** Patients with acute ST-elevation myocardial infarction were divided into three groups based on their admission plasma glucose level. Three patients were lost to follow up, and eight patients died within the follow-up period.

A total of 83 consecutive patients were allocated to the normal, median, or high admission plasma glucose group for glucose levels of < 7.8 mmol/L, between 7.8 and 11.1 mmol/L, or ≥11.1 mmol/L, respectively (Figure 1). All included patients remained in their allocated groups during the follow-up period.

### Interventions

All patients were administered clopidogrel (300 mg) and aspirin (300 mg) 10–40 min before catheterization and underwent routine primary percutaneous coronary intervention (PCI). Coronary angiography (CA) was performed using the standard Seldinger technique, in order to demonstrate the absence of coronary collaterals in the risk region and to locate the site of occlusion in the infarct relative artery. The number of blocked coronary vessels and the location of stenosis were recorded. Revascularization was performed by direct stenting. The number of stents implanted, and the type of stent used (bare-metal or drug-eluting) were also recorded. Patients were then monitored in the coronary care unit (CCU) for 1–3 days. Discharge medications for each patient included aspirin, β-blockers, statins, angiotensin-converting enzyme (ACE) inhibitors, or angiotensin-receptor blockers (ARBs).

### Data collection and follow up

Each patient’s hospitalization data were recorded in accordance with the protocol. On hospital admission, 3 mL blood was withdrawn from an antecubital vein. Samples were collected into prechilled evacuation tubes containing ethylenediaminetetra-acetic acid (EDTA). The samples were centrifuged at 2000 rpm in a BECKMAN CS-15R centrifuge (Beckman Coulter, Brea, CA, USA) for 10 min and stored at -70°C until the assay was performed. sFas plasma levels were measured with a sandwich enzyme-linked immunosorbent assay (ELISA) kit: Human Fas (CD95)/TNFRSF6 Quantikine ELISA Kit (R&D Systems Inc., Minneapolis, MN, USA).

Patients were followed up by telephone from the day of acute STEMI admission until death, emigration, or the end of study on September 8, 2013. The predefined primary outcome was all-cause mortality. Data concerning whether the patient died, when death occurred, and the cause of death were registered. Meanwhile, the numbers of rehospitalizations for CA were retrospectively analyzed at the end of the follow-up period.

### Statistical analysis

Categorical data are presented as proportions. Groups were compared with chi-square analyses. Continuous data are presented as the mean ± standard deviation (SD). Comparison of the groups by analysis of variance (ANOVA) was followed by Newman-Keuls multiple comparison analysis to determine differences between individual groups. A P value < 0.05 for two-tailed tests was considered significant. Kaplan–Meier analysis was performed to estimate the 8 years follow-up mortality. SPSS version 18.0 software was used for statistical calculations (SPSS Inc., Chicago, IL, USA).

## Results

### Patient characteristics

A total of 83 eligible consecutive patients with primary acute STEMI were included between April 2005 and December 2005. Patients were divided into three groups based on their admission plasma glucose level. There were 33, 24, and 26 patients in the normal, median, and high glucose groups, respectively (Figure 1). There were no significant differences in the discharge medication or the proportion of patients with hypertension or other cardiovascular risk factors. The difference in glycosylated hemoglobin (HbA1C) level among the groups was significantly different (P < 0.05). Triglycerides were significantly higher in the high glucose group compared to the normal glucose group (P < 0.05) (Table 1).

**Table 1:** Baseline characteristics

	**Normal glucose group**	**Median glucose group**	**High glucose group**
	**(n = 33)**	**(n = 24)**	**(n = 26)**
Age (y)	58 ± 11	61 ± 10	59 ± 11
Male (%)	28 (84.8%)	20 (83.3%)	18 (69.2%)
BMI (kg/m^2^)	25.3 ± 1.8	25.0 ± 2.7	25.7 ± 2.5
**Medical history**			
Hypertension, n (%)	20 (60.6%)	19 (79.2%)	21 (80.8%)
Previous diabetes, n (%)	2 (6.1%)	6 (25.0%)	12 (46.2%)^*^
Previous angina, n (%)	5 (15.1%)	3 (12.5%)	3 (11.5%)
Atrial fibrillation, n (%)	0 (0%)	0 (0%)	1 (3.8%)
Current smoker, n (%)	21 (63.3%)	15 (62.5%)	20 (76.9%)
**Biochemical data**			
Cholesterol (mmol/L)	4.81 ± 0.85	4.46 ± 1.13	4.80 ± 1.21
LDL-cholesterol (mmol/L)	2.78 ± 0.68	2.57 ± 0.75	2.78 ± 0.83
HDL-cholesterol (mmol/L)	1.02 ± 0.22	0.95 ± 0.26	0.96 ± 0.21
Triglycerides (mmol/L)	1.31 ± 0.65	1.27 ± 0.83	1.98 ± 1.42^*^
hsCRP (mg/L)	3.2 ± 2.8	2.3 ± 1.6	3.7 ± 4.5
CKMB (IU/L)	137.9 ± 96.9	145.7 ± 85.5	100.6 ± 89.5
Creatinine (μmol/L)	91.48 ± 29.34	85.04 ± 26.74	96.15 ± 19.31
GFR (ml/min)	78.22 ± 24.28	79.43 ± 27.45	70.90 ± 15.85
HbA1C (%)	4.5 ± 1.1	5.3 ± 1.2^†^	7.6 ± 2.0^*, #^
Admission plasma glucose level (mmol/L)	6.3 ± 0.9	9.1 ± 0.9^†^	14.5 ± 3.4^*, #^
**Clinical aspects of STEMI**			
Ischemia time			
Symptom-to-enrollement, min	355 ± 162	340 ± 151	346 ± 180
Symptom-to-ballon, min	419 ± 159	409 ± 149	414 ± 183
Infarct site: Anterior	51.5%	54.2%	57.7%
Infarct related artery			
LAD, n (%)	18 (55%)	11 (46%)	13 (50%)
LCX, n (%)	3 (9%)	3 (13%)	3 (11%)
RCA, n (%)	12 (36%)	10 (42%)	10 (38%)
Number of diseased vessels			
1 coronary vessel (%)	23 (70%)	17 (71%)	16 (62%)
2 coronary vessels (%)	8 (24%)	6 (25%)	8 (31%)
3 coronary vessels (%)	2 (6%)	1 (4%)	2 (7%)
Number of stent implanted	1.48 ± 0.51	1.58 ± 0.50	1.50 ± 0.51
Stent type			
Bare-metal stent, (%)	44%	39%	38%
Drug-eluting stent, (%)	56%	61%	62%

*BMI* body mass index; *HDL* high-density lipoprotein; *hsCRP* high-sensitivity C-reactive protein; *LDL* low-density lipoprotein; *CKMB* creatine kinase isoenzyme MB; *GFR* glomerular filtration rate; *HbA1C* glycosylated hemoglobin;

*P < 0.05 for the high glucose group versus the normal glucose group.

^#^P < 0.05 for the high glucose group versus the median glucose group.

^†^P < 0.05 for the median glucose group versus the normal glucose group.

All patients were accepted for primary PCI and monitored in the CCU for 1–3 days. Discharge medications for each patient included aspirin, β-blockers, statins, ACE inhibitors, or ARBs. Three patients were lost to follow up, eight patients died within the 8-year follow-up period, and seven of the eight patients suffered a cardiovascular death (Figure 1).

### Correlation between the admission plasma glucose and sFas levels

**Figure 2:**
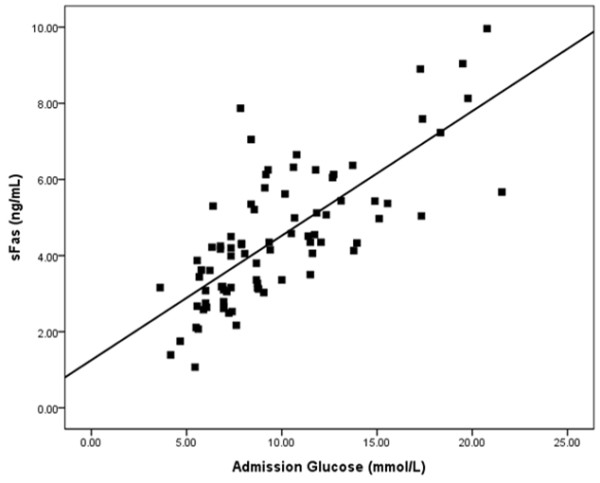
**Correlation between the admission glucose level and the sFas level.** We found that sFas levels increased in accordance with improvement in the admission glucose level. The correlation coefficient *R* is 0.747, the *R*^2^ is 0.559, with a regression line equation of Y = 1.225 + 0.327X (P < 0.05).

The sFas levels of all patients were determined by ELISA. There was a significant difference among the three groups (Table 2). The sFas levels were higher in the high glucose group (5.87 ± 1.70 ng/mL; 95% confidence interval [CI], 5.14-6.51) than in the normal glucose group (3.07 ± 0.93 ng/mL; 95% CI, 2.74-3.40) (P < 0.05), and they were elevated in the median glucose group (4.84 ±1.38 ng/mL; 95% CI: 4.25-5.42) compared to the normal glucose group (3.07 ± 0.93 ng/mL) (P < 0.05). There was no significant difference in sFas level between the high and median glucose groups. The scatter plot of the admission plasma glucose and sFas levels shows a positive association; the correlation coefficient (*R*) was 0.747, *R*^2^ was 0.559, and the regression line equation was Y = 1.225 + 0.327X (P < 0.05) (Figure 2).

**Table 2:** Soluble fas concentrations

	**Normal glucose group**	**Median glucose group**	**High glucose group**
	**(n = 33)**	**(n=24)**	**(n=26)**
Soluble fas (ng/mL)	3.07 ± 0.93	4.84 ± 1.38^#^	5.87 ± 1.70^*^

*P < 0.05 for the high glucose group versus the normal glucose group;

^#^P < 0.05 for the median glucose group versus the normal glucose group.

### Follow up after acute STEMI

**Figure 3:**
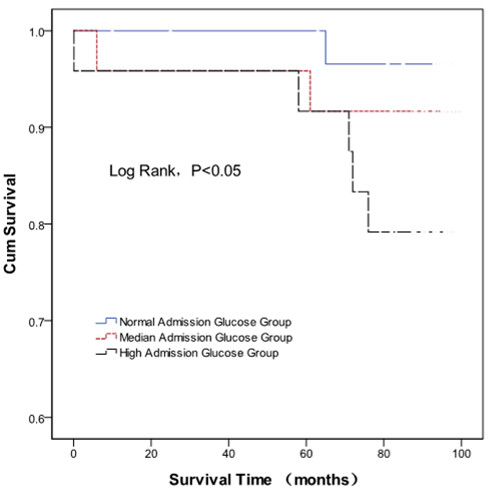
**Kaplan-Meier survival curves of acute ST-elevation myocardial infarction patients with different admission plasma glucose levels.** Mortality was higher in the high glucose level group than the normal glucose level group (P < 0.05).

All patients were followed up for an average of 89 ± 20 months. The normal, median, and high admission plasma glucose level groups were followed for 90 ± 21 months, 90 ± 20 months, and 87 ± 21 months, respectively. The normal and high admission plasma glucose groups lost one and two patients to follow up, respectively. In the normal, median, and high glucose groups, one, two, and five patients died, which corresponded to mortality rates of 3.0%, 8.3%, and 19.2%, respectively. There was a significant difference between the normal and high plasma glucose groups (P < 0.05). The Kaplan-Meier survival curves are shown in Figure 3.

All patients rehospitalized for CA were retrospectively analyzed at the end of the follow-up. In total, 11 patients were rehospitalized for CA, owing to angina or re-infarction. The numbers of patients receiving in-stent re-stenosis in the normal, median, and high glucose groups, were one, two, and two, respectively, and the rates of in-stent re-stenosis in these three groups were 25%, 50%, and 67%, respectively.

## Discussion

It was reported that nearly two-thirds of patients with cardiovascular disease suffer from abnormal glucose metabolism [9,10]. In-hospital measurements of HbA1C and admission plasma glucose may be useful as early markers of long-standing glucometabolic disturbance [11]. A prior diabetes diagnosis and admission blood glucose concentration are associated with adverse outcomes after an AMI. Gholap et al. [12] found that the admission glucose level was strongly associated with mortality in all presentations of AMI, irrespective of whether a diabetes diagnosis had previously been established. However, the follow-up periods in previous reports were brief [8,12]; there are few long-term follow-up studies of acute STEMI patients with high admission plasma glucose levels.

In the current study, patients with acute STEMI were divided into three groups based on their admission plasma glucose level. All patients were followed up for an average of 89 ± 20 months. The mortality rate was higher in the high admission plasma glucose group than in the normal group (19.2% vs. 3.0%, respectively, P < 0.05). Kaplan-Meier survival curves depict the high mortality rate in the high admission glucose level group and suggest that prognosis is poor for acute STEMI patients with an admission plasma glucose level higher than 11.1 mmol/L.

It has been suggested that high levels of plasma glucose at hospital admission is positively associated with rates of in-stent re-stenosis and poor prognosis [13,14]. In the present study, we did not repeat CA for evaluating the rates of in-stent re-stenosis at follow-up, because it is an invasive method that requires in-hospital treatment and increases treatment costs. In addition, patients without symptoms were unwilling to accept repeat CA. The rate of in-stent re-stenosis was a secondary rather than primary outcome in clinical follow up. In the present study, patients were followed up for the primary end-point outcomes, (all-cause mortality and cardiovascular mortality), in order to identify the relationship between plasma glucose concentrations and the apoptosis marker sFas upon admission and at the first end-point outcome after acute STEMI.

There is a growing body of studies on the relationship between high plasma glucose levels and heart health [15-19]. Most previous studies have found that a high glucose level induces or aggravates myocardial injury. High ambient glucose disturbs the cell cycle, increases DNA damage, delays endothelial cell replication, and causes excessive cell death [19]. Several studies from different laboratories demonstrate that high glucose mediates endothelial cell to chondrocyte transition in human aortic tissue, and selectively triggers apoptosis in cultured endothelial cells [15,19]. During apoptosis, or programmed cell death, cells gradually lose their normal morphology and undergo genomic DNA degradation that is not associated with necrosis. The existing data indicate that apoptosis plays an important role in myocardial ischemia in both humans and animal models [20].

Fas was identified as a valuable biomarker of the physiological response to ischemia [4,5]. Patients with AMI who experience hypoxia undergo apoptotic myocardial cell death that is associated with increased serum concentrations of sFas. This correlation of cell death with serum concentrations of Fas and sFas in AMI patients suggests that Fas-mediated cardiomyocyte apoptosis is involved in disease pathology [21].

Kageyama et al. [22] found that the exposure of human coronary artery endothelial cells to a high glucose environment significantly increased Fas expression. Coronary vessels from type 2 diabetic mice displayed appreciable expression levels of tumor necrosis factor receptor 1 (TNF-R1) and Fas [22]. The present study demonstrated that sFas levels were significantly higher in the high admission glucose level group compared to the normal glucose level group (P < 0.05), and sFas concentration was positively associated with admission plasma glucose level (P < 0.05).

### Limitations

A limitation of this study is that it was performed in a small patient sample at a single center. We were unable to divide the subjects into subgroups, which may have influenced the statistical power of our analyses. There were fewer data for in-stent re-stenosis, as the number of repeat CA cases at follow-up were not included. Further studies are needed to expand the sample size, including the number of repeat CA cases at follow-up, and to confirm these findings.

## Conclusion

The results of this study indicate that in patients with acute STEMI, sFas concentration is positively associated with the admission plasma glucose level. Fas apoptosis was increased in patients with high glucose levels at admission glucose, and these patients had higher mortality on long-term follow up compared to those with normal glucose levels at admission.

## Abbreviations

ACE: Angiotensin-converting enzyme; AMI: Acute myocardial infarction; ARBs: Angiotensin-receptor blockers; CA: Coronary angiography; CCU: Coronary care unit; CI: confidence interval; CKMB: Creatine kinase isoenzyme MB; cTnI: Cardiac troponin I; EDTA: Ethylenediaminetetra-acetic acid; ELISA: Enzyme-linked immunosorbent assay; hsCRP: High-sensitivity C-reactive protein; PCI: Percutaneous coronary intervention; STEMI: ST-elevation myocardial infarction.

## Competing interests

The authors declare that they have no competing interests.

## Authors’ contributions

ZL conceived and designed the study and revised the manuscript. ZG, ZL and LXL acquired and analyzed data. CJ drafted the first version of the manuscript. HYP provided general advice on the clinical data analysis. All authors interpreted the data, critically revised the manuscript for important intellectual content, and approved the final version of the manuscript for submission.
